# Cost-effectiveness of implementing performance-based financing for improving maternal and child health in Ethiopia

**DOI:** 10.1371/journal.pone.0305698

**Published:** 2024-07-15

**Authors:** Mideksa Adugna Koricho, Girmaye Deye Dinsa, Nelisiwe Khuzwayo

**Affiliations:** 1 School of Nursing and Public Health, Discipline of Public Health, University of KwaZulu-Natal, Durban, South Africa; 2 Oromia Health Bureau, Addis Ababa, Ethiopia; 3 Department of Global Health and Population Harvard T. H. Chan School of Public Health, Fenot Associates, Addis Ababa, Ethiopia; 4 Department of Public Health and Health Policy, College of Health Sciences, Haramaya University, Harar, Ethiopia; 5 Department of Health Policy and Management, Jimma University, Jimma, Oromia, Ethiopia; OHE: Office of Health Economics, UNITED KINGDOM

## Abstract

**Introduction:**

Performance Based Financing (PBF) supports realization of universal health coverage by promoting bargaining between purchasers and health service providers through identifying priority services and monitoring indicators. In PBF, purchasers use health statistics and information to make decisions rather than merely reimbursing invoices. In this respect, PBF shares certain elements of strategic health purchasing. PBF implementation began in Ethiopia in 2015 as a pilot at one hospital and eight health centers. Prior to this the system predominantly followed input-based financing where providers were provided with a predetermined budget for inputs for service provision. The purpose of the study is to determine whether the implementation of PBF is cost-effective in improving maternal and child health in Ethiopia compared to the standard care.

**Methods:**

The current study used cost-effectiveness analysis to assess the effects of PBF on maternal and child health. Two districts implementing PBF and two following standard care were selected for the study. Both groups of selected districts share common grounds before initiating PBF in the selected group. The provider perspective costing approach was used in the study. Data at the district level were gathered retrospectively for the period of July 2018 to June 2021. Data from health service statistics were transformed to population level coverages and the Lives Saved Tool method used to compute the number of lives saved. Additionally for purpose of comparison, lives saved were translated into discounted quality-adjusted life years.

**Results:**

The number of lives saved under PBF was 261, whereas number of lives saved under standard care was 194. The identified incremental cost per capita due to PBF was $1.8 while total costs of delivering service at PBF district was 8,816,370 USD per million population per year while the standard care costs 9,780,920 USD per million population per year. QALYs obtained under PBF and standard care were 6,118 and 4,526 per million population per year, respectively.

**Conclusions:**

The conclusion made from this analysis is that, implementing PBF is cost-saving in Ethiopia compared to the standard care.

**Limitations of the study:**

- Due to lack of district-level survey-based data, such as prevalence and effects on maternal and child health, national-level estimates were used into the LiST tool.
- There may be some central-level PBF start-up costs that were not captured, which may have spillover effects on the existing health system performance that this study has not considered.
- There may be health statistics data accuracy differences between the PBF and non-PBF districts. The researchers considered using data from records as reported by both groups of districts,

## 1. Introduction

Though global health expenditure has been raising faster than global economic growth during the last five years [1] the health financing landscape of countries is facing many challenges in moving towards universal health coverage (UHC). This includes financial barriers that hinder households from utilizing healthcare. Additionally, health system of many countries are characterized by inefficiencies and wastage of resources that account for 20% to 40% of the health resources[1], which could be highly attributed to the financing arrangements of the health system [2].

Likewise, the global challenges and health systems in Sub-Sahara African (SSA) countries share the health financing pitfalls of the systems that follow input-based financing in which systems pay pre-determined amount of resource for providers. One of the major gaps in this financing approach is that health systems do not incentivize providers to render adequate and quality services [3]. By 2017, over 810 women per day passed away because of causes that can be prevented in relation to pregnancy and delivery worldwide, with low and lower-middle-income countries (LMICs) accounting for 94% of all maternal deaths. Similarly, in 2016, LMICs experienced 15.6 million excess deaths from 61 conditions, of which 8.6 million were avoidable, only if expanded service coverage is accompanied by investments in high-quality health systems [4,5].

In Ethiopia, as of 2017, the estimated maternal mortality ratio stood at 401 deaths per 100,000 live births. This translates to approximately 33 women losing their lives each day due to complications arising from pregnancy and childbirth. This high maternal mortality is related to challenges such as inadequate availability of skilled birth attendants, inadequate access to emergency obstetric care, and lack of essential maternal health services. These challenges contribute to increased risks and complications during pregnancy and delivery, leading to tragic outcomes for many women in Ethiopia [6]. During the same year under five children mortality in the country stood at 67 per 1000 live births which is also attributed to limited access to essential health services [7,8].

LMICs made efforts to improve the efficiency, effectiveness, and quality of health services during the last couple of decades. These include reforming the organization of health systems and introducing different financing mechanisms [9]. Performance-based financing (PBF) is among the financing initiatives many countries adopted. PBF is an instrument that links financing to predetermined results or targets, with payment made upon verification that the agreed-upon results/outputs have been achieved [10]. PBF aims to minimize challenges related to the poor performance of a health system. As a tool, PBF supports creating better, more inclusive, and more accessible health services. Evidence from SSA countries indicates that PBF can strengthen core health system functions[11]. This could be expressed through improved quantity and quality of health services supplied for the community [12].

PBF payments depend explicitly on the efforts providers exert to achieve specific pre-established targets verified by performance indicators and achievements adjusted for some quality measures [13]. PBF helps to streamline the focus of health system objectives when purchasers, funders, or the government could choose payment indicators that are supposed to reflect the national burden of disease and health priority [14–17]. As PBF shares certain elements of strategic health purchasing, is viewed as a way of moving towards strategic purchasing and strengthening efforts to meet universal health coverage goals [16,18]. Many countries implemented PBF to improve the delivery of priority health interventions. It is also implemented in many countries with the prime purpose of improving maternal and child health services, as maternal and child mortality is a challenge that presents a substantial burden [19]. Evidence indicates that implementation of this program contributed to enhancements in maternal and child health services and outcomes [20,21] as well as increased job satisfaction and decreased attrition of the health workforce [22].

Generally, there is increasing evidence indicating that PBF schemes can increase service use and quality of service [23]. PBF results are mixed, with little evidence confirming that it is a pro-poor strategy because unintended consequences can affect PBF outcomes [24,25]. There is also limited evidence on the cost-effectiveness of PBF as an implementation strategy [9] as its implementation requires the allocation of additional resources compared to other financing arrangements such as input-based financing. Therefore, knowing the status of cost-effectiveness of PBF is important to support policy makers when making choices to this financing arrangement in health.

Thus, this cost-effectiveness evaluation examines whether the implementation of PBF is more or less cost-effective in improving maternal and child health in Ethiopia compared with the input-based financing. The study uses data from a PBF pilot program in the Oromia region in Yabello town and Yabello District. This study seeks to inform decisions about PBF implementation not only for Ethiopia but in applications to other low-income country settings. This article is presented in accordance with the Consolidated Health Economic Evaluation Reporting Standards (CHEERS) reporting checklist *(S1 File).*

## 2. Methods

### Study setting

Ethiopia introduced PBF in 2015, becoming one of the few SSA countries to do so. The Oromia regional administration initiated this program with financial and technical assistance from CORD AID, an internationally functioning Netherlands-based nongovernmental organization, and the Embassy of the Kingdom of the Netherlands. The program was created to promote healthcare coverage and quality, with a focus on maternal and child health services. Yabello town and its nearby rural administration, Yabello district, were chosen for this purpose. There were eight health centers and one hospital in the districts under this financing approach. During the design of this program, CORDAID’s national office was functioning as a purchaser for the PBF pilot, where its local branch served as a verifier. Data for this study were gathered retrospectively for the period of July 2018 to June 2021. The data was first accessed by September 2021.

### The comparator

For this study the comparator districts were chosen based on their structural resemblance to the intervention districts such as similarity in socioeconomic status in which majority of the population lives in rural set-up, livelihood of the community is mainly pastoralist and geographically all districts under the study exists in one geographic administration.

Both groups of selected districts share common grounds before PBF is initiated in the selected group in terms of key health outcomes and resource allocation mechanisms. After the introduction of PBF, health facilities in the intervention group receives payment attributed to health service quantity and quality. Contrary to the intervention districts, non-PBF districts (comparator) did not get any additional cash above and beyond their regularly allotted budgets or any overt actions to enhance the health system.

### Research design

The study estimates the cost-effectiveness of PBF compared to non-PBF implementation (standard care). The costing approach used was a health service provider perspective costing approach that did not consider societal and individual cost perspectives. The study’s goal was to assess the influence of PBF on healthcare delivery in four (4) districts over the three years (2019–2021) among which two districts are PBF districts and two are non-PBF (standard care) districts.

Data at the district level were collected from relevant organizations. District finance offices and CORDAID provided the cost information. At the same time district health offices provided the information on health statistics for priority maternal and child health services.

### Cost assessment

A provider perspective costing approach is taken into account in the cost-effectiveness study. Financial costs data were gathered for the analysis. We concentrated on the expenditures of the districts classified as PBF and non-PBF/standard care groups in this analysis. Salary and related costs, operational costs, drug and supply costs, PBF implementation support expenses by CORDAID, and incentives paid to district health offices and health facilities from July 2018 to June 2021 were gathered and included in the analysis for the PBF groups. Similarly, salaries and related costs, operational costs, medicine and supply costs were gathered and included in the study for the non-PBF group. Utilizing the midyear exchange rate over the years, costs accrued in birr were converted to USD (US$). During those three years, the average USD to Ethiopian Birr exchange rates were 28.02, 31.6, and 39.3. The costs were finally reported after adjusting to the inflation. We distributed pooled expenses of operations, capacity-building, verification, monitoring, and evaluation costs that CORDAID spent for the PBF districts. Due to the uniformity in how the activities were carried out, the districts participating in PBF share the aforementioned expenditures equally. Finally, the costs estimated for each group of districts were rescaled by the population covered by the PBF and non-PBF districts to determine costs per capita per year.

### Measure of effectiveness

Given the goals of Ethiopia’s PBF program, evaluating hypothesized increases in the use of maternal and child health services was the main emphasis of measuring effectiveness. We acquired the statistical data from district health offices in PBF and non-PBF areas to assess the impact on utilization. The list of health interventions on which data was collected included:

- Contraceptive use
- Post-abortion case management
- Tetanus toxoid vaccination
- Syphilis detection and treatment during pregnancy
- Iron supplementation in pregnancy
- Clean birth environment and clean cord care
- Antibiotics for maternal sepsis
- Assisted vaginal delivery
- Neonatal resuscitation
- Cesarean delivery
- Blood transfusion
- Vitamin A supplementation
- Zinc supplementation
- Households protected from malaria using insecticide-treated bed nets/indoor residual spraying
- Vaccinations for bacillus calmette–guérin (BCG), Polio, diphtheria, pertussis, and tetanus (DPT), pneumococcal, rotavirus, and measles
- Kangaroo mother care
- Case management of neonatal sepsis/pneumonia
- Diarrhea treatment
- Pneumonia treatment
- Treatment for acute and moderate malnutrition
- Prevention of mother-to-child transmission of HIV/AIDS
- Ante-retroviral treatment for children

Health statistics information collected for the health interventions listed above was transformed into coverages using available country estimates *(*S1 Table*)*. The calculated coverage levels were fed into the Lives Saved Tool (LiST), which is a component of the well-known OneHealth platform for unified costing and impact calculation. This tool was used to convert health service coverage into the number of lives saved [26], which was later converted to quality of life that serves as an additional or supplementary outcome of interest for this study.

The LiST model uses the international public health literature to assess the efficacy of interventions [27,28]. The use of this model in Ethiopia implies that the assumption would also hold true at the sub-national level. To compare the population number of the PBF group with the non-PBF/standard care group for this study, we considered essential parameters from Ethiopian data preloaded in the LiST program, including demographic data. The LiST model calculates the impact on live saved of a single intervention using the change in coverage multiplied by the efficacy of the intervention. For every interventions in either preventive or curative categories the impact calculation is performed by LiST. The LiST tool generates the number of lives saved for mothers, infants under the age of one, and children under the age of five based on included maternal and child health interventions.

As a supplementary analysis we converted the lives saved into discounted quality-adjusted life years (QALYs) using the following formula[29]:

$$QALY_{s\;gained}=Q*\frac{1-e^{-r(L_{a})}}{r}$$

Where: Q is the average quality of life if one survives, r is the annual discount rate, and La is life expectancy at the age of death of a, estimated from Ethiopia’s life table [30], and e is 2.718. Q was estimated from the disease burden in Ethiopia [31] using the following equation:

$$\left(1-\frac{d-a}{h*p}\right)$$

Where: d is a disability, a is adjusted life years due to morbidity, h is healthy life expectancy, and p is population size.

We estimated the total QALYs gained by multiplying the number of lives saved by QALYs gained per case. This was done separately for infants under one year old, children under five years old, and mothers later added together to generate total QALYs. The total QALYs gained were then rescaled by the population covered under the PBF and non-PBF districts to estimate QALYs gained per capita.

### Cost-effectiveness analysis

After discounting life years at 3%[32] and adjusting the cost for inflation, we calculated costs per live saved for both groups of interest of the study.

### Sensitivity analysis

We undertook a deterministic sensitivity analysis across key model parameters changing each parameter at a time up to ±20% of the base case value. We also conducted probabilistic sensitivity analysis (PSA) using Monte Carlo simulation of 1000 times to evaluate parameter uncertainty and yield a 95% CI around the result assuming skilled birth attendant as a proxy indicator for the PBF programme. Appropriate distributions were assigned for the PSA. Accordingly, Gamma distributions were specified for costs, normal distribution for live saved, and Beta distributions for delivery service[33]. Finally, we presented the results as cost–effectiveness scatter plot.

### Patient and public involvement

No patients or public members participated in the design, implementation, or reporting of this study. The decision-makers who will receive the research findings include the regional health bureau, the ministry of health, and health managers at the study sites.

## 3. Ethics approval

This study was ethically cleared and approved by the Biomedical Research Ethics Committee (BREC) of the University of KwaZulu-Natal (Ref: BREC/00002678/2021), South Africa, and the Institutional Ethics Review Committee of Oromia Health Bureau (Ref: BEFO/UBTFH/H6/2), Ethiopia. Written informed consent was obtained from the districts during data collection.

## 4. Inclusivity in global research’

Additional information regarding the ethical, cultural, and scientific considerations specific to inclusivity in global research is included in the *(S2 File).*

## 5. Results

### Cost of implementing PBF versus standard care

The table below shows the implementation costs for the PBF program and standard care (input-based financing). From July 2018 to June 2021, the total cost of executing the PBF program in the two intervention districts was $3,895,482, or $13.9 per capita per year. Total PBF expenditure was $826,905 from total spending or $3.0 per capita per year. From July 2018 to June 2021, the total cost at districts under standard care was $6,295,742 or $15.9 per capita per year *(*Table 1*)*.

**Table 1:** Expenditures of PBF and non-PBF districts (2019–2021).

Expenditure category	PBF				Non-PBF			
	2019	2020	2021	Ave	2019	2020	2021	Ave
**Direct staff cost (ETB)**	28,729,605	25,292,344	27,409,311	27,143,753	51,999,472	49,617,284	48,641,287	50,086,015
**Operational Cost (ETB)**	4,411,729	3,460,950	3,838,392	3,903,690	17,770,154	12,758,592	9,196,872	13,241,873
**Drug and supply (ETB)**	1,559,921	2,340,182	2,006,394	1,968,832	4,465,331	3,703,244	3,284,113	3,817,563
**Total PBF payment (ETB)**	9,322,151	7,872,104	9,632,105	8,942,120	-	-	-	-
**Total Expenditure (ETB)**	44,023,406	38,965,579	42,886,201	41,958,395	74,234,957	66,079,121	61,122,272	67,145,450
**Catchment Population**	90,716	93,170	95,624	93,170	128,535	132,313	136,090	132,313
**Per capita Expenditure (ETB)**	485	418	448	450	578	499	449	507
**Total Expenditure (USD)**	1,571,142	1,233,088	1,091,252	1,298,494	2,649,356	2,091,111	1,555,274	2,098,581
**Per capita Expenditure (USD)**	17.3	13.2	11.4	13.9	20.6	15.8	11.4	15.9
**PBF Expenditure (USD)**	332,696	249,117	245,092	275,635	-	-	-	-
**Per capita-PBF Expenditure (USD)**	3.7	2.7	2.6	3.0	-	-	-	-

The breakdown of yearly PBF program expenses revealed that $1.6 per capita (54%) was paid as PBF incentive payments to health facilities. In comparison, $1.4 per capita (46%) was the administrative cost for the purchasing agency (CORDAID) and payments provided to regulators such as district and zonal health offices. The staff costs ($0.47), general administration ($0.45), capacity building for staff at health facilities ($0.002), technical support ($0.2), travel ($0.04), and capital items ($0.12) made up these yearly per capita operating expenditures. The wage and related costs were estimated to be US$ 9.6 for the PBF group and US$ 11.8 for the input-based financing, while the medicine and supply costs per capita per year were estimated at US$ 0.6 for the PBF group and US$ 0.9 for the input-based financing.

### Impacts on lives saved and QALYs gained

The lives of 11 mothers and 250 children under the age of five per million people per year, i.e a total of 261 lives were saved arising from the PBF’s improvements to service delivery. The lives of 10 mothers were spared under the standard care, and 184 children under the age of five were saved per million people annually, for a total of 194 lives.

Furthermore, expressing this in QALYs, the number of QALYs gained from improved service delivery under PBF was 6,118, and under standard care, it was 4,526 per million people per year.

### Cost-effectiveness result of the analysis

The computed cost per live saved for PBF is 14,915 USD and for standard care is 32,455 USD. Furthermore, the cost per QALYs for PBF is 637 USD and for standard care is 1,391 USD. As a result, this analysis shows that putting PBF into practice is preferable as a cost minimization strategy over the standard care (S1 Fig). According to the PSA result, probability of PBF to continue being cost minimization strategy over the standard care is 53%.

## 6. Discussion

With an emphasis on maternal and child healthcare, this study estimated the value of PBF and standard care in terms of cost and effectiveness. The findings revealed that the standard care in Ethiopia costs USD 501,922.95 per million per year or USD 15.9 per capita, while the PBF program costs USD 441,038.79 per million per year or USD 13.9 per capita. Apart from regular input-based financing, the expenditures attributed to PBF alone were $3 per person per year, significantly less than the most current evidence from SSA [21,34,35] except for Zimbabwe which was $2.32 [36]. As the evidence indicates, costs per capita in Ethiopia for implementing PBF is much lower than that of Malawi [$6.46].

The allocation of yearly per capita costs for the PBF program reveals that over half, specifically 54% of the total, is designated for health facilities as incentive payments. This allocation signifies a highly favorable situation for health service providers, as it enables them to acquire additional resources to enhance the quality of their services. In comparison to other countries, the percentage of PBF payments allocated as health facility incentives in Malawi was 33.6% [34], and Tanzania, it was 22% [37]. Thus, the current allocation demonstrates a more advantageous scenario in terms of incentivizing health facilities through the PBF program.

There is evidence from several low-income countries that PBF can improve the delivery of health interventions that improve quality of life, particularly for mothers and children. Due to the implementation of results-based financing in Benin, the facility-based delivery rate has increased from 19% in 2006 to 57% in 2011 [38]; there has also been an increase in the number of pregnant women in Burundi receiving anti-tetanus vaccinations [39]. According to research conducted in Rwanda, PBF also caused a protective association with specific types of malnutrition (wasting) in children under five [40]. It also led to higher rates of modern methods of contraception being used in PBF-implemented areas (60.3%) than in the control group (40.6%), as identified from the Kumbo district of Cameroon [41].

There might be the case since PBF health facilities outperform traditionally funded/resourced health facilities in terms of antenatal care (ANC) visits, outpatient department attendance, deliveries at health facilities, children who have received all recommended vaccinations, and the number of family planning service users[42]. According to the review done across five countries, PBF enhanced facility delivery, ANC utilization, or ANC care quality in certain nations among poor women [43]. In general, PBF was found to lower under-five mortality as a contributing factor to bettering health outcomes.

There is a study that shows PBF can improve health outcomes. The study found that there was a two percentage point decrease in under-five mortality but that this decrease was only significant for children of mothers who had above-median wealth (2.7 percentage points) and education (2.1 percentage points) [44].

The outcome of this study demonstrates that Ethiopia’s PBF program, compared to standard care i.e. traditional financing method, is a cost saving strategy for enhancing maternal and child health in saving more lives. Similarly in other PBF implementation areas the health benefits were greater than of the standard care. Cognizant to this, there is positive findings from Zimbabwe, where PBF is cost-effective at $1,166 per QALYs (1.21 times the per capita GDP), and in Zambia, where PBF is cost-effective at $837 per QALYs, (0.48 times the per capita GDP). Similar findings have been identified from Malawi, where PBF was a cost-effective strategy to enhance perinatal and maternal healthcare and outcomes [36]. The incentive-based program also able to increase attended deliveries and lower maternal mortality at an acceptable cost according to findings from a cost-effectiveness analysis of a voucher program combined with obstetrical quality improvements from Uganda[45].

## 7. Conclusion

The additional cost of implementing PBF in Ethiopia is 3 USD per capita, which is significantly less expensive than in other SSA nations. According to this study, PBF is a cost saving strategy for financing health facilities to enhance health of mother and children. The deciding factor that led to this finding was that PBF costs $14,915 per live saved where the standard care costs $32,455 per live saved.

In general, this study demonstrates PBF as a cost saving strategy, suggesting that there could be a space to accommodate PBF in the Ethiopia’s healthcare financing system.

## Supporting information

S1 File CHEERS checklist. (DOCX)

S2 File Inclusivity in global research. (DOCX)

S1 Table Health statistics data (2019–2021). (XLSX)

S1 Fig Cost-effectiveness scatter plot for PBF relative to standard care. (TIF)
