# Supplementary figures and images for: Cost-effectiveness of implementing performance-based financing for improving maternal and child health in Ethiopia
Source: PLoS One. 2024 Jul 15;19(7):e0305698. doi: 10.1371/journal.pone.0305698 (PMC11249211; doi:10.1371/journal.pone.0305698)

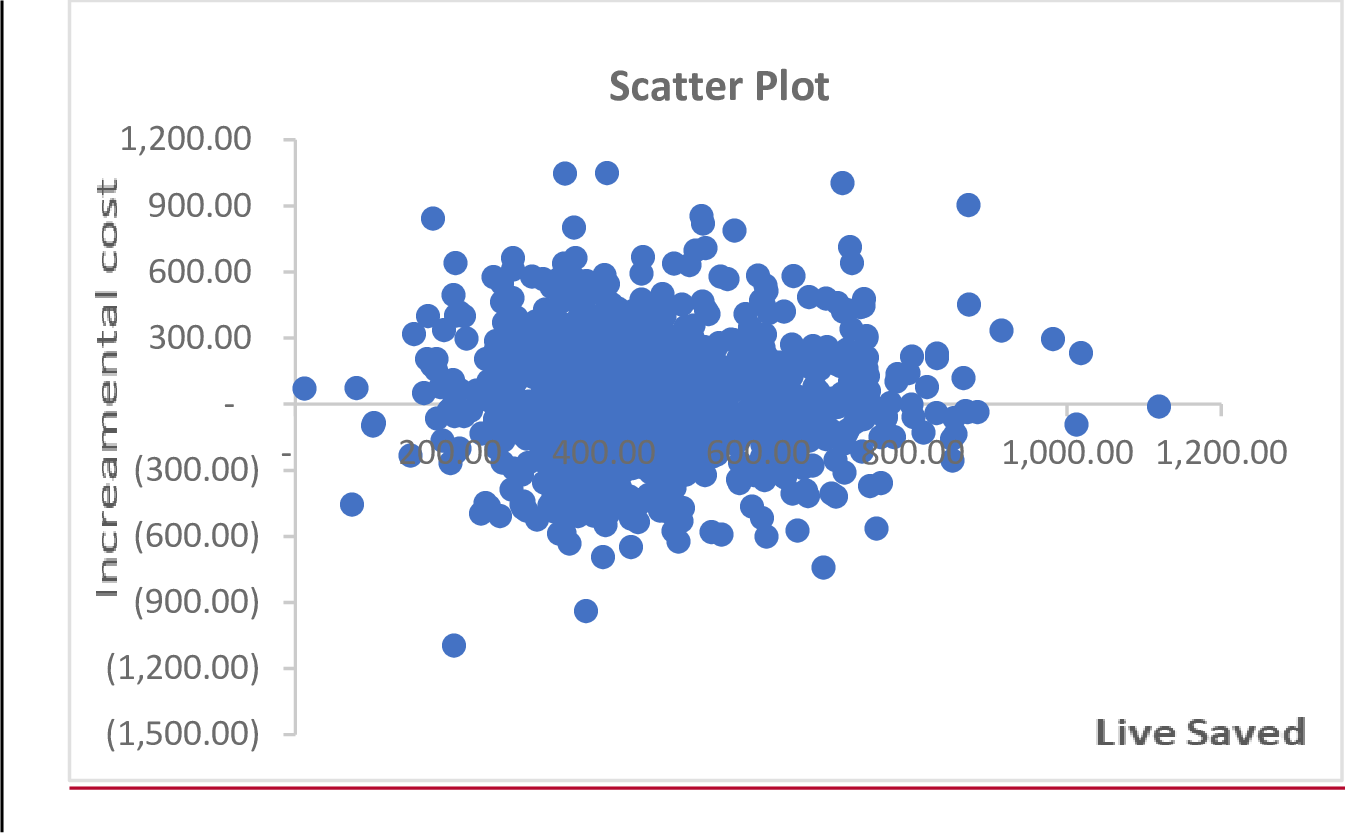

Supplement: S1 Fig — (TIF) [file pone.0305698.s004.tif]
